# Comparative Analysis of Gut Microbiota Responses to New SN-38 Derivatives, Irinotecan, and FOLFOX in Mice Bearing Colorectal Cancer Patient-Derived Xenografts

**DOI:** 10.3390/cancers17132263

**Published:** 2025-07-07

**Authors:** Katarzyna Unrug-Bielawska, Zuzanna Sandowska-Markiewicz, Magdalena Piątkowska, Paweł Czarnowski, Krzysztof Goryca, Natalia Zeber-Lubecka, Michalina Dąbrowska, Ewelina Kaniuga, Magdalena Cybulska-Lubak, Aneta Bałabas, Małgorzata Statkiewicz, Izabela Rumieńczyk, Kazimiera Pyśniak, Michał Mikula, Jerzy Ostrowski

**Affiliations:** 1 Department of Genetics, Maria Sklodowska-Curie National Research Institute of Oncology, 02-781 Warsaw, Poland; 2 Department of Gastroenterology, Hepatology and Clinical Oncology, Centre of Postgraduate Medical Education, 02-781 Warsaw, Poland

**Keywords:** irinotecan, FOLFOX, colorectal cancer patient-derived xenografts, stool microbiota, stool metabolites

## Abstract

Background: Symbiotic gut microbiota can enhance cancer therapy efficacy, while treatment-induced dysbiosis may reduce effectiveness or increase toxicity. Our preclinical study compared the anticancer effects and impact on fecal microbiota and metabolites of two water-soluble SN-38 derivatives (BN-MePPR and BN-MOA), with those observed after treatment with Irinotecan, and the FOLFOX regimen in NOD scid gamma mice bearing patient-derived colon adenocarcinoma xenografts (CRC PDX). Methods: Five individual experiments with Irinotecan and its derivatives and eight individual experiments with FOLFOX were conducted using eight CRC PDX models. Chemotherapeutics were administered intraperitoneally 4–5 times at 5-day intervals. Fecal samples were collected before and after treatment. Microbiota composition was analyzed by 16S rRNA gene (V3–V4 regions) sequencing. Mass spectrometry was used to quantify short-chain fatty acids (SCFAs) and amino acids (AAs). Results: All treatments significantly inhibited tumor growth versus controls. However, no significant changes were observed in gut microbiota α- and β-diversity between treated and untreated groups. Tumor progression in controls was associated with increased abundance of *Marvinbryantia*, *Lactobacillus*, *Ruminococcus*, and [Eubacterium] *nodatum* group. FOLFOX-treated mice showed increased *Marvinbryantia*, *Bacteroides*, and *Candidatus Arthromitus*, and decreased *Akkermansia*. No distinct taxa changes were found in the Irinotecan or derivative groups. SCFA levels remained unchanged across groups, while BN-MePPR, BN-MOA, and Irinotecan all increased AA concentrations. Conclusions: Contrary to earlier toxicological data, these findings indicate a relatively limited impact of the tested chemotherapeutics on the gut microbiome and metabolome, emphasizing the importance of research method selection in preclinical studies.

## Simple Summary

The bacteria living in our intestines, known as gut microbiota, may affect the benefits of cancer treatments. Some cancer therapies may disturb this delicate balance, possibly reducing their effectiveness or causing more side effects. In this study, we tested chemotherapy treatments in mice bearing colorectal cancer patient-derived xenografts, searching for a relationship between tumor growth and both the structure of gut microbiota and profiles of short-chain fatty acids and amino acids. However, while the treatments successfully slowed tumor growth, they had only minor effects on gut bacteria and selected fecal molecules. These findings suggest that certain chemotherapy drugs may not disturb the gut environment in animal cancer models as much as previously thought. This research could help guide future studies and improve the methodology of cancer treatments in preclinical studies.

## 1. Introduction

Irinotecan (CPT11), a semi-synthetic and water-soluble analog of the plant alkaloid camptothecin (CPT), is a topoisomerase I (TOP1) inhibitor used widely (either individually or in combination with other cytotoxic drugs such as 5-fluorouracil (5-FU) or oxaliplatin) to treat colon cancer, non-small-cell lung cancer, pancreatic cancer, gastric cancer, and other cancer types [1]. It is listed on the WHO Model List of Essential Medicines as one of the most important medications required by a basic healthcare system [2]. Irinotecan is converted to its active metabolite, 7-ethyl-10-hydroxycamptothecin (SN-38) by hepatocyte carboxylesterases (CES1 and CES2), which hydrolyze the ester and amide bonds present in some pharmaceutical products [3]. SN-38 is hundreds to thousands of times more toxic than Irinotecan until enzymatically converted to SN-38 glucuronide (SN-38G) by uridine diphosphate glucuronosyltransferases (UGT) in the liver; the nontoxic product is then moved to the intestine via the bile [4]. However, β-glucuronidases secreted by gut bacteria can reconvert the excreted, inactive metabolite SN-38G back to the toxic SN-38 [5]. SN-38 is toxic to all proliferating cells, resulting in major dose limits and potentially life-threatening side effects in many patients (mainly delayed diarrhea, autoimmune suppression, and hepatotoxicity).

Topoisomerases relax the torsional strain within supercoiled DNA during replication and transcription by cleaving one strand, creating a nick, and enabling rotation around the uncleaved strand; this is followed by ligation of the broken strand and restoration of the DNA duplex [6,7]. SN-38 binds to the DNA-TOP I complex and forms a stable ternary complex that prevents religation of DNA strands and interferes with the moving replication fork; this induces replication arrest and causes lethal double-strand DNA breaks, the central mechanism underlying the antitumor activity of Irinotecan [8]. Although the water-soluble nature of SN-38 prevents its clinical applications [9], new water-soluble derivatives, including 7-ethyl-9 (N-methylphenethyl)methyl-10-hydroxycamptothecin(BN-MePPR) and 7-ethyl-9 (N-morpholinyl)metyl-10-hydroxycamptothecin (BN-MOA), have a unique mechanism based on spontaneous alkylation of aromatic elements within DNA molecules and are the most effective blocker of Topo I activity [10]. Consequently, these compounds are more effective in killing breast, leukemia, colon, and lung cancer cells than the clinically used Irinotecan [11]. However, before they can be tested in early-phase clinical trials, preclinical studies assessing their activity and toxicity are needed. As a result, the activity of these compounds against breast, leukemia, colon, and lung cancer cell lines in vitro is magnitudes greater than that of the clinically used Irinotecan (an API of Pfizer’s CAMPTO formulation) [11]; however, before these derivatives can be tested in early-phase clinical trials, preclinical studies assessing their activity and toxicity are needed.

The richness, diversity, and composition of the gut microbiota are modulated by host genotype, age and sex, lifestyle, diet, physical activity, sanitation, and many other environmental factors. Microbial products and microbiota-related metabolites such as short-chain fatty acids (SCFAs), amino acids (AAs), secondary bile acids, and lipopolysaccharides are considered to be potential modulators of the development and progression of various human pathologies, including cancer [12]. A healthy, symbiotic microbiome can enhance the effectiveness and reduce the toxicity of certain cancer therapies, through activating certain cancer drugs, improving their absorption, or enhancing their interaction with the immune system. In turn, disruptions to this balance, known as dysbiosis, can impair drug efficacy and increase harmful side effects [13]. Side effects of Irinotecan, 5-FU, cyclophosphamide, oxaliplatin, gemcitabine, and methotrexate associated with intestinal dysbiosis have been observed in animal and clinical studies [14,15]; however, it is still not clear whether chemotherapeutics cause microbial dysbiosis directly or indirectly [16].

The goal of this preclinical study was to compare the anticancer activity, as well as the impact on the fecal microbiota and selected metabolites, of BN-MePPR- and BN-MOA in immunodeficient NOD scid gamma (NSG) mice bearing human colon adenocarcinoma patient-derived xenografts (PDX) with those observed after treatment with Irinotecan or a combination of 5-FU, oxaliplatin, and folinic acid (i.e., the FOLFOX regimen).

## 2. Materials and Methods

### 2.1. Tumor Engraftment

The CRC PDX model, based on NU/J mice, was developed at the Maria Sklodowska-Curie National Institute of Oncology [17]. The PDXs resected from NU/J mice (P3–P7) were cut into sections (10–20 mm^3^) and frozen in media containing 50% DMEM, 40% FBS, and 10% DMSO. After ice-breaking, tumor pieces were placed subcutaneously on the lateral sides of NSG mice (6–10 weeks old) (*n* = 2–4). When the tumor volume reaches 1000–1500 mm^3^, tumor tissues are resected and implanted in the left side of the NSG mice, and the experiments begin. Animals are randomly assigned to treated and non-treated (control) groups when tumors exceed 80–150 mm^3^.

### 2.2. Administration of Chemotherapy

Five independent experiments were carried out with Irinotecan and the two SN-38 derivatives (BN-MePPR and BN-MOA) and eight independent experiments with FOLFOX. The groups were composed of 4–8 mice. FOLFOX chemotherapy regimen consists of oxaliplatin (6 mg/kg) + 5-FU (50 mg/kg) + folic acid (90 mg/kg). Irinotecan (40 mg/kg), BN-MePPR (40 mg/kg), BN-MOA (40 mg/kg), and FOLFOX were administered intraperitoneally (IP) at intervals of 5 days. The control groups received 0.9% saline vehicle treatment. Mice were measured, and tumor size was measured by a caliper before taking the drugs. The tumor volume is calculated using the following formula: (length × width × width)/2. Mice in a group were sacrificed when the size of most tumors in control groups reached about 1200 mm^3^ or earlier, if the mice showed signs of distress. All experiments were carried out in accordance with EU Directive 2010/63/EU and approved by the 2nd Animal Experiments Ethics Committee (WAW2/014/2020), which was held in Warsaw on 29 January 2020.

The stool samples were collected from each mouse before and after the experiments and stored at 80 °C until they were used for 16S rRNA-Seq metagenomics and metabolic methods. Bacterial DNA was isolated from fecal samples using a mini kit QIAamp Fast DNA Stool (Qiagen, Hilden, Germany), as described earlier [18]. The quality and quantity of the extracted DNA were evaluated by measuring optical density using NanoDrop 2000/2000c spectrophotometers (Thermo Fisher Scientific, Carlsbad, CA, USA) and fluorometric methods using Qubit dsDNA HS Assay Kits (Thermo Fisher Scientific). The preparation of a library of V3 and V4 variables of the 16S rRNA gene of bacterial cells was performed according to the preparation protocol for the 16S metagenomic sequence library preparation in the Illumina platform (Illumina, Inc., San Diego, CA, USA). Sequences were obtained using an Illumina MiSeq system with 2 × 300 bp parallel ends.

SCFA and AA metabolites were extracted and prepared for estimations as described previously [19]. The gas chromatography analysis of the extracts was performed with the Agilent 7000D Triple Quadrupole Mass Spectrometer, connected to the 7890 gas chromatography (GC) system and the G4513A auto sampler (Agilent Technologies, Santa Clara, CA, USA). The VF-5ms column (30 m, 0.25 mm, 0.50 m) was used for analysis. Mass spectrometry (MS) data are collected in a complete scanning mode (*m*/*z* 15–650, frequency 4.9 scans/s). The data were analyzed using Mass Hunter software (Agilent Technologies).

### 2.3. Statistical Methods

Differences in tumor size between the treated and control groups in the chemotherapy experiments were evaluated using an independent-samples *t*-test or the Mann–Whitney U-test. A two-sided *p* < 0.05 was considered significant. All statistical analyses were performed using GraphPad, v8.2 (GraphPad Software, San Diego, CA, USA).

For RNA-Seq, version 1.26 [20] of the DADA2 24 pipeline was used for reading error correction, amplicon sequence variant identification, and chimeric reading identification and removal. The taxonomy assignment was carried out by Mothur version 1.43 [21] with SILVA (version 1.138 26) [22]. The diversity index (Shannon and Chao1) was calculated using 1000 bootstrap samples using version 3.0 of the iNEXT R package. Significant differences between groups were identified by the Mann–Whitney U test or by a mixed effect linear model (as implemented in the lmerTest package version 3.1 [23]). Distances (β-diversity) were calculated with the robust Aitchison method using the vegan package version 2.6-4. A significant group was identified with the adonis2 function. LINDA [24] identified variants of the abundant amplicon sequence (as implemented in Microbiome Stat version 1.1 package). The difference in the relative abundance of AAs and SCFAs was determined by selecting the function “Multiple *t* Tests (and Non-Parametric Tests)—one per row” in GraphPad Prism v10.1.1.

## 3. Results

**Figure 1:**
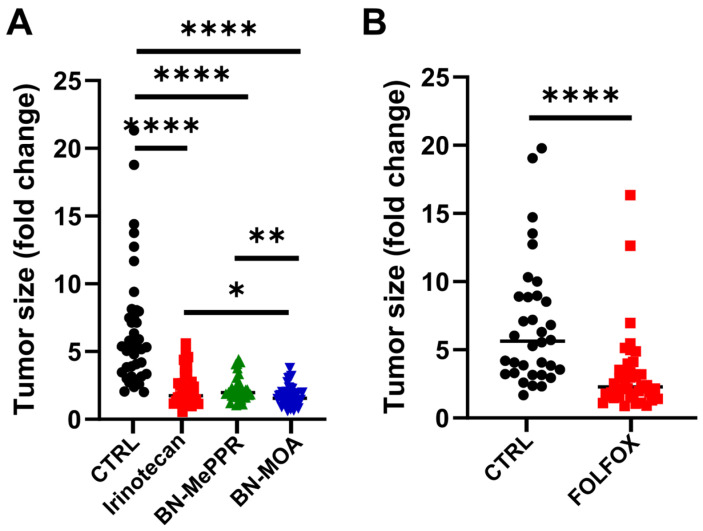
Therapeutic effects of chemotherapeutics administered via 4–5 intraperitoneal injections at 5-day intervals to CRC PDX-bearing mice. Effects were assessed by estimating fold changes in tumor volume from the start of treatment to the end of the experiment for (**A**) Irinotecan and its derivatives (BN-MePPR and BN-MOA) and (**B**) FOLFOX regimen compared to controls. The results from separate experiments using the same compounds were pooled to give the following sample sizes: irinotecan group, *n* = 33; BN-MePPR group, *n* = 28; BN-MOA group, *n* = 28, corresponding control group, *n* = 40; FOLFOX group, *n* = 37 and the corresponding control group, *n* = 36. Statistical significance: * *p* < 0.05; ** *p*  <  0.01; **** *p*  <  0.0001.

Results pertaining to tumor size, body weight, microbiome, diversity, and taxonomic and metabolomic abundance are presented as fold changes from the time of treatment initiation to the end of the experiment. To increase the power of statistical testing, the results obtained from individual groups were combined. As shown in Figure 1, both Irinotecan and FOLFOX inhibited tumor growth significantly when compared with the vehicle-treated controls. Irinotecan and BN-MePPR reduced tumor growth to the same level, while BN-MOA reduced growth even more significantly.

**Figure 2:**
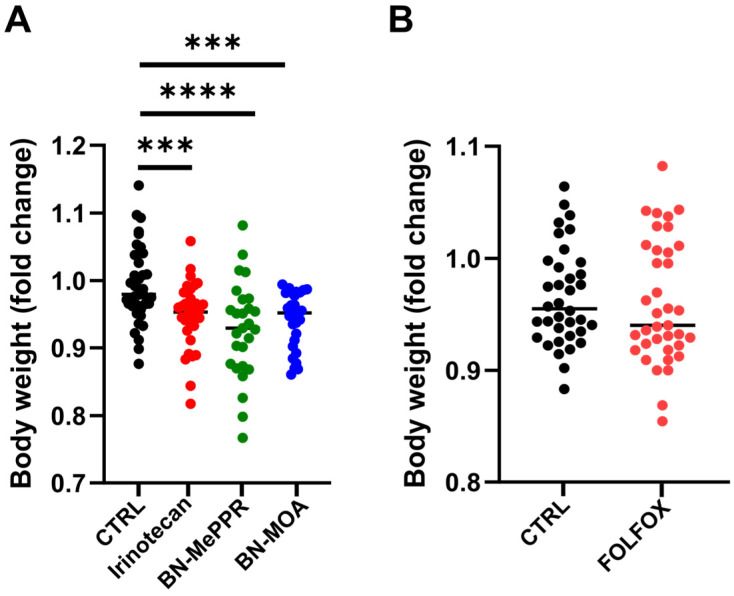
Fold changes in mouse body weight from the start to the end of the experiments: (**A**) Irinotecan, BN-MePPR, and BN-MOA treatment groups compared to controls. (**B**) FOLFOX-treated group compared to controls. The results from separate experiments using the same compounds were pooled to give the following sample sizes: irinotecan group, *n* = 33; BN-MePPR group, *n* = 28; BN-MOA group, *n* = 28, corresponding control group, *n* = 40; FOLFOX group, *n* = 37 and the corresponding control group, *n* = 36. Statistical significance: *** *p*  <  0.001; **** *p*  <  0.0001.

Animals were weighed at weekly intervals to an accuracy of 0.1 g. Figure 2 shows the fold changes in body weight from the start to the end of the experiments; there was no difference between the control and FOLFOX-treated groups (Figure 2B). By contrast, treatment with Irinotecan and the two derivatives resulted in a significant reduction in body weight in each of the study groups compared with the respective control group; there was no significant difference between the two study groups (Figure 2A).

**Figure 3:**
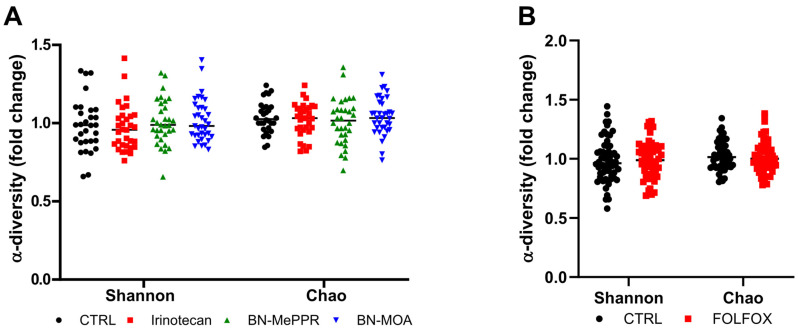
Fold changes in α-diversity indices (Shannon and Chao) in fecal samples collected at the beginning and end of the experiments: (**A**) Shannon and Chao1 indices in mice treated with Irinotecan, BN-MePPR, or BN-MOA compared to controls. (**B**) Shannon and Chao1 indices in mice treated with FOLFOX compared to controls. The results from separate experiments using the same compounds were pooled to give the following sample sizes: irinotecan group, *n* = 33; BN-MePPR group, *n* = 28; BN-MOA group, *n* = 28, corresponding control group, *n* = 40; FOLFOX group, *n* = 37 and the corresponding control group, *n* = 36.

α-diversity (analyzed by calculating the Shannon index) is a marker of richness and evenness, and Chao1 is a marker of bacterial richness. These indices do not differ between studies and control groups from the beginning to the end of the experiment (Figure 3).

**Figure 4:**
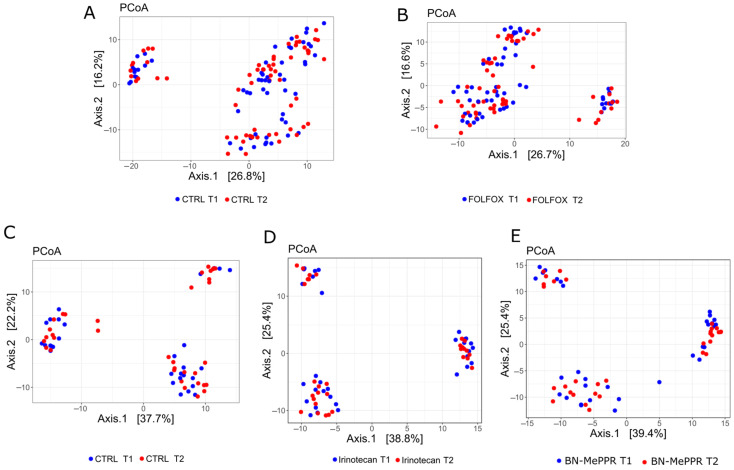
Principal coordinate analysis (PCoA) using the Euclidean metric in fecal samples collected at the beginning and end of the experiments. Each dot represents a single sample. The results from separate experiments using the same compounds were pooled to give the following sample sizes: (**A**) the corresponding control group, *n* = 36, (**B**) FOLFOX group, *n* = 37, (**C**) corresponding control group, *n* = 40, (**D**) irinotecan group, *n* = 33, (**E**) BN-MePPR group, *n* = 28; BN-MOA group, *n* = 28.

The β-diversity of the bacterial structure analyzed by principal coordinate analysis (PCoA) of Bray–Curtis distances did not differentiate the study groups from the control groups at the initiation of treatment or at the end of the experiments. Instead, there was a variation in the β-diversity between different experiments carried out at different times (Figure 4).

Taxonomic analysis identified four genera (*Marvinbryantia, Lactobacillus, Ruminococcus*, and [Eubacterium] *nodatum* group) in control mice that were underrepresented at the end of the experiment (T1) compared with the beginning of the experiment (T0) (Table 1). By contrast, pairwise comparison of T0 and T1 in FOLFOX-treated mice identified two genera (*Marvinbryantia*, *Bacteroides*) that were underrepresented, one genus (*Candidatus Arthromitus*) that tended to be underrepresented (adj. *p* = 0.068), and one genus (*Akkermansia*) that tended to be overrepresented (adj. *p* = 0.068) at the end of the experiments (Table 2). No differential taxa were found in the Irinotecan- and derivative-treated groups.

**Figure 5:**
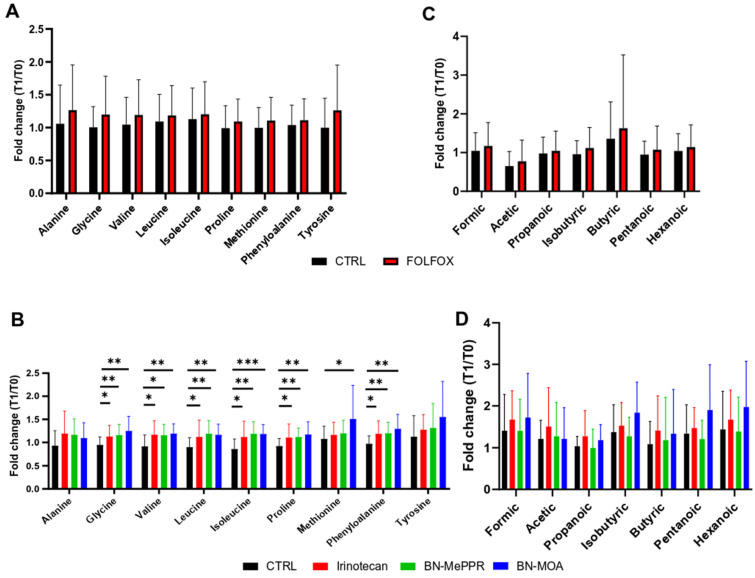
Fold change in the relative abundance of amino acids (AAs) and short-chain fatty acids (SCFAs) in fecal samples collected at the beginning and end of the experiments: (**A**,**B**) AAs in FOLFOX-treated mice (**A**) and in mice treated with Irinotecan, BN-MePPR, or BN-MOA (**B**), compared to respective controls. (**C**,**D**) SCFAs in FOLFOX-treated mice (**C**) and in mice treated with Irinotecan, BN-MePPR, or BN-MOA (**D**), compared to respective controls. Data from separate experiments using the same compounds were pooled to give the following sample sizes: irinotecan group, *n* = 33; BN-MePPR group, *n* = 28; BN-MOA group, *n* = 28, corresponding control group, *n* = 40; FOLFOX group, *n* = 37 and the corresponding control group, *n* = 36. Statistical significance: * *p* < 0.05; ** *p* < 0.01; *** *p* < 0.001.

Nine AAs and seven SCFAs were analyzed in fecal samples collected at the beginning and end of the experiments. While differences in the fold changes in the abundance of SCFAs and AAs between T0 and T1 were not significant in the FOLFOX- and Irinotecan/derivate-treated groups, or in the FOLFOX-treated groups, respectively, treatment with Irinotecan or BN-MePPR led to an increase in the fold changes of six AAs (glycine, valine, leucine, isoleucine, proline, methionine, and phenylalanine), and treatment with BN-MOA led to an increase in the fold change of seven AAs (glycine, valine, leucine, isoleucine, proline, methionine, and phenylalanine) (Figure 5) between T0 and T1.

## 4. Discussion

Irinotecan, 5-FU, and oxaliplatin damage both cancer cells and normal cells indiscriminately, leading to severe side effects such as chemotherapy-induced gastrointestinal toxicity, which is accompanied by microbial and metabolic changes [14,25,26]. Here, we aimed to compare the anticancer effects, as well as effects on the gut microbiota and metabolite composition, of two derivatives of SN-38 (BN-MePPR and BN-MOA) with those of Irinotecan and FOLFOX, one of the standard first-line regimens for the treatment of advanced CRC [27]. Chemotherapeutics were administered to xenograft-bearing mice 4 or 5 times at 5-day intervals.

Treatment efficacy was determined by calculating fold changes in tumor volume between the start and end of each experiment. The volume of the grafted PDXs in control (untreated) mice increased gradually from 80–150 mm^3^ at the beginning of the experiment to 1000–2000 mm^3^ 3–4 weeks later; as expected, all of the chemotherapeutics studied inhibited tumor growth. Among groups of mice treated with Irinotecan and its derivatives, BN-MOA reduced tumor growth significantly more than the two other drugs, which showed similar antitumor activity. While the FOLFOX regimen did not cause body weight loss by the end of the experiment, mice receiving Irinotecan or its derivatives lost a significant amount of body weight.

16S rRNA-Seq metagenomics analyses showed no significant difference in either the α- or β-diversity of the gut flora of the treated and control groups. However, the gut microbiome is a living ecosystem that naturally fluctuates and, therefore, some changes might be temporary or within the range of normal variation. Furthermore, many microbes can perform similar functions, so the loss of one species might not be detrimental if others can compensate. In a consequence, minimal changes in diversity can be misleading, as a treatment might shift the composition of the microbiome, even if the overall diversity remains relatively unchanged. When a treatment reduces the abundance of harmful bacteria but also reduces the abundance of some beneficial bacteria, this may result in a net change in composition without a significant change in diversity.

While diversity is a valuable metric, it is not the only factor to consider when evaluating the effects of a treatment on the microbiome. Analyzing both diversity and composition, along with functional changes, is essential for a comprehensive understanding.

Taxonomic analysis at the genus level identified increased relative abundance of four taxa (*Marvinbryantia*, *Lactobacillus*, *Ruminococcus*, and [Eubacterium] *nodatum* group) in control mice at the end of experiments, which was likely related to the progression of tumor growth. *Marvinbryantia* is a cellulose- and methylcellulose-degrading bacterial genus belonging to the family *Lachnospiraceae*, whose fecal abundance is higher in 6-week-old mice than in 72-week-old mice [26]. In APCmin/+ mice, microcystin-LR toxin promotes the progression of colon tumors and impairs intestinal barrier function, accompanied by enrichment of *Marvinbryantia* within the gut microbiota [28]. *Marvinbryantia* is also one of the four taxa enriched in patients and mice that correlated with a decrease in tumor growth [29].

Irinotecan and its two derivatives reversed the increased abundance of all four genera observed in the control groups, and FOLFOX treatment reversed the increase in all but Marvinbryantia; however, while no changes in taxa abundance were observed in Irinotecan- and derivative-treated mice, FOLFOX increased the abundance of *Candidatus Arthromitus* and *Bacteroides*. As reported previously [30], *Candidatus_Arthromitus* is one of five bacterial genera that dominate the mouse jejunum, and is a predominant taxon that increased in abundance after a 6-day treatment with 5-FU (50 mg/kg/day). Medicinal plants (*Astragalus mongholicus Bunge* and *Curcuma aromatica Salisb*), which exert therapeutic effects on digestive tract tumors, decrease the abundance of *Candidatus_Arthromitus* in tumor-bearing mice [31]. Contrary to our findings, a recent study [31] reported that 5-FU decreased the abundance of Bacteroides, which play a protective role against breast and colon cancer development both in vitro and in vivo [32,33,34], and *Bacteroides fragilis* protects against colon tumorigenesis in a mouse model of colitis-associated colon cancer [34]. The beneficial genus *Akkermansia*, which prevents or ameliorates metabolic diseases and enhances antitumor immune responses [35], was identified as a potential gastric and colorectal cancer biomarker in humans [36,37,38]. In our study, we found that FOLFOX decreased the abundance of *Akkermansia* significantly in fecal samples obtained at the end of the experiments.

Previous toxicological studies show that treating rats for 3 days with CPT11 (125 mg/kg body weight) increases the abundance of intestinal *Enterobacteriaceae* spp. and Clostridium cluster XL [39], and that a 3-day treatment of mice with CPT11 (150 mg/kg) decreases the richness of the gut microbiota by increasing the abundance of the phylum Proteobacteria and the families *Porphyromonadaceae* and *Mogibacteriaceae* [40]. A single dose of irinotecan (200 mg/kg) administered to rats significantly reduced the abundance of *Bifidobacterium* spp., *Lactobacillus* spp., *Bacteroides* spp., and *Staphylococcus* spp., and increased that of *Escherichia* spp., *Clostridium* spp., *Enterococcus* spp., *Serratia* spp., and *Staphylococcus* spp., peaking at between 72 and 144 h post-treatment [41,42,43]. Mice injected with 5-FU (25 mg/kg/d) and Irinotecan (25 mg/kg/d) for 4 days to induce intestinal mucositis displayed reduced α-diversity of the fecal microbiota, clearly separated α-diversity, increased abundance of the genera *Lactobacillus* and *Bacteroidetes*, and decreased abundance of *Norank_f_Muribaculaceae, Alloprevotella*, and *Prevotellaceae_UCG_001* [44].

While cross-sectional studies provide valuable insights, longitudinal research is crucial for a comprehensive understanding of the gut microbiome’s role in health and disease, especially when considering the long-term effects and dynamic nature of these microbial communities. Longitudinal studies can help disentangle the complex interplay between the host and its gut microbiome and can assess the stability of the gut microbiome over time. This is particularly important in conditions where dysbiosis is a hallmark. However, as in many gut microbiome studies, a significant limitation of this study is the lack of focus on long-term effects and longitudinal data.

Another important limitation of our study is the use of NSG mice, which, although essential for the engraftment and propagation of patient-derived xenografts, lack functional T, B, and NK cells. This profound immunodeficiency alters host–microbiota interactions and may affect the gut microbial response to chemotherapeutic agents. In immunocompetent hosts, the immune system plays a key role in shaping microbial composition, regulating mucosal barrier integrity, and modulating systemic responses to microbial metabolites. Therefore, the absence of immune surveillance in NSG mice may limit the translational relevance of our microbiota-related findings. While our results provide valuable insights into the direct effects of chemotherapy on the gut ecosystem in a controlled setting, future studies using humanized or partially reconstituted immune models may help bridge the gap between preclinical and clinical observations.

It is generally accepted that disruption of the gut microbiota can alter its metabolic function [45]. The intestinal microbiota is the primary source of bacteria that generate SCFAs, a group of fecal microbial metabolites that impact gut barrier integrity and permeability, modulate brain-induced intestinal gluconeogenesis, regulate histone deacetylase activity, and influence glucose and lipid metabolism as well as innate and adaptive immune responses [46]. Indeed, SCFAs serve as energy sources, improve intestinal barrier integrity, and exert anti-inflammatory effects [47,48]. In addition, bacterial metabolic processes in distal parts of the colon may be related to the availability of AAs [49].

Modulation of SCFA production linked to intestinal inflammation is a function of the gut–microbiome–metabolome axis [10,50,51]. In agreement with the relatively small impact of the tested chemotherapeutic agents on the fecal microbiota in our tumor mouse model, we found that treatment with neither FOLFOX nor Irinotecan/SN-38 derivatives altered the relative abundance of fecal SCFAs between the treated and untreated (control) groups. By contrast, while FOLFOX treatment did not change fecal levels of any of the AAs studied, Irinotecan and BN-MePPR increased the fold change in glycine, valine, leucine, isoleucine, proline, methionine, and phenylalanine levels from the beginning to the end of the experiment, whereas BN-MOA increased that of glycine, valine, leucine, isoleucine, proline, methionine, and phenylalanine, compared with the control groups.

Previous studies report that the influence of dietary fiber on CPT-11 toxicity is mediated partially by cecal production of butyrate [52]. Feeding a high-fiber diet to mice with mucositis modulates the composition of the gut microbiota and its metabolic ability to produce SCFAs [2], and induction chemotherapy decreases the level of fecal SCFAs in acute myeloid leukemia patients [53]. In patients with CRC, Capecitabine decreases fecal levels of the SCFAs valerate and caproate, although chemotherapy-induced toxicity was not significantly associated with SCFA levels [54]. In apolipoprotein E-deficient (ApoE^−/−^) mice, aspirin increases the levels of propionic acid, valeric acid, isovaleric acid, and isobutyric acid, which correlates with an improved immuno-inflammatory profile [55]. Another study showed that while the reduction in two-cycle CPT-11/5-FU therapy toxicity by non-digestible carbohydrates did not correlate with stimulation of specific bacterial taxa, it correlated strongly with the concentration of cecal butyrate [52].

Animal and human studies show that SCFAs, especially butyrate, suppress tumor growth and cancer cell metastasis, and act synergistically with anticancer drugs [56,57]. In addition, there is evidence for a relationship between AA metabolism and the effects of chemotherapeutics. Cisplatin, tamoxifen, and doxorubicin affect tyrosine and alanine levels in breast cancer cell lines, which might contribute to drug resistance [58,59]. The efficacy of platinum-based chemotherapy is regulated by effector T cells via cysteine and cysteine metabolism, and high levels of intracellular glutathione in tumor cells lead to reduced cisplatin accumulation in the nucleus, thereby increasing tumor cell resistance to cisplatin-based chemotherapy [60]. The microbiota-derived tryptophan metabolite indole-3-acetic acid (3-IAA) is enriched in the serum of pancreatic ductal adenocarcinoma (PDAC) patients who respond to treatment, and oral administration of 3-IAA increases the efficacy of chemotherapy in humanized gnotobiotic mouse models of PDAC [61]. Chemotherapy treatment of breast cancer patients increases fecal levels of glycine, valine, glutamate, threonine, and alanine [62]. Finally, a study in a CRC orthotopic xenograft mouse model showed that a high-salt diet significantly reduces the efficacy of FOLFOX, which correlates with the composition of the gut microbiota and levels of tryptophan metabolites [63].

## 5. Conclusions

Our study investigated the efficacy of prolonged treatment of tumor-bearing mice with Irinotecan, two SN-38 derivatives (BN-MePPR and BN-MOA), and FOLFOX chemotherapy, as well as their impact on the composition of gut microbiota and selected fecal metabolites. In contrast to previous toxicological studies [64,65] that investigated intestinal microbiota in the context of intestinal mucositis in rats or mice treated with higher doses of Irinotecan and 5-FU, albeit over a shorter time period, we observed relatively moderate changes in the composition of the gut microbiome. We did not detect any changes at fecal levels of SCFAs. The only apparent difference was in AA levels in mice treated with Irinotecan, BN-MePPR, and BN-MOA (but not FOLFOX). Although treatment with Irinotecan/SN-38 derivatives caused a significant decrease in body weight, there were no other side effects such as diarrhea. Therefore, although a previous study highlighted careful selection of the methods used in preclinical studies [66,67], the mouse model used herein did not confirm an advantage of the newly established inhibitors over the commonly used drug Irinotecan.

## Figures and Tables

**Table 1:** Differential taxa identified in control mice between treatment initiation and the end of the experiments (results are compiled from each separate experiment; *n* = 46).

Phylum	Class	Order	Family	Genus	Fold Change	Adjusted *p*-Value
Firmicutes	Clostridia	Lachnospirales	*Lachnospiraceae*	*Marvinbryantia*	0.35	4.22 × 10^−5^
Firmicutes	Bacilli	Lactobacillales	*Lactobacillaceae*	*Lactobacillus*	0.40	2.98 × 10^−4^
Firmicutes	Clostridia	Oscillospirales	*Ruminococcaceae*	*Ruminococcus*	0.48	2.05 × 10^−3^
Firmicutes	Clostridia	Peptostreptococcales-Tissierellales	*Anaerovoracaceae*	*[Eubacterium] nodatum group*	0.48	1.09 × 10^−2^

**Table 2:** Differential taxa identified in FOLFOX-treated mice from the time of treatment initiation to the end of the experiments (results are compiled from each separate experiment, *n* = 37).

Phylum	Class	Order	Family	Genus	Fold Change	Adjusted *p*-Value
Firmicutes	Clostridia	Lachnospirales	*Lachnospiraceae*	*Marvinbryantia*	0.39	0.0016
Bacteroidota	Bacteroidia	Bacteroidales	*Bacteroidaceae*	*Bacteroides*	0.67	0.0056
Verrucomicrobiota	Verrucomicrobiae	Verrucomicrobiales	*Akkermansiaceae*	*Akkermansia*	1.99	0.068
Firmicutes	Clostridia	Clostridiales	*Clostridiaceae*	*Candidatus Arthromitus*	0.59	0.068

## Data Availability

The datasets presented in this study can be found in online repositories. The names of the repositories and accession numbers can be found below: https://www.ncbi.nlm.nih.gov/bioproject/1268737 and 1199764 (accessed on 28 May 2025).

## Abbreviations

The following abbreviations are used in this manuscript:

5-FU	5-fluorouracil
AAs	amino acids
BN-MePPR	7-ethyl-9-(N-(N-methylpiperazinyl)methyl-10-hydroxycamptothecin
BN-MOA	7-ethyl-9-(N-morpholinyl)metyl-10-hydroxycamptothecin
CES	carboxylesterase
CPT	plant alkaloid camptothecin
CRC	colon adenocarcinoma
NOD	non-obese diabetic mouse
NSG	NOD scid gamma mouse
PDX	patient-derived xenografts
SCFAs	short-chain fatty acids
SN-38	7-ethyl-10-hydroxycamptothecin
TOP1	topoisomerase I
UGT	uridine diphosphate glucuronosyltransferases

## References

[1] Rothenberg M.L. (2001). Irinotecan (CPT-11): Recent Developments and Future Directions–Colorectal Cancer and Beyond. Oncologist.

[2] Gallotti B., Galvao I., Leles G., Quintanilha M.F., Souza R.O., Miranda V.C., Rocha V.M., Trindade L.M., Jesus L.C.L., Mendes V. (2021). Effects of Dietary Fibre Intake in Chemotherapy-Induced Mucositis in Murine Model. Br. J. Nutr..

[3] Merali Z., Ross S., Paré G. (2014). The Pharmacogenetics of Carboxylesterases: *CES1* and *CES2* Genetic Variants and Their Clinical Effect. Drug Metab. Drug Interact..

[4] Takakura A., Kurita A., Asahara T., Yokoba M., Yamamoto M., Ryuge S., Igawa S., Yasuzawa Y., Sasaki J., Kobayashi H. (2012). Rapid Deconjugation of SN-38 Glucuronide and Adsorption of Released Free SN-38 by Intestinal Microorganisms in Rat. Oncol. Lett..

[5] Wu R., Chen J., Jia Y., Chen Y., Li Y., Yan R. (2023). Fecal Microbial SN-38G Metabotypes in Colorectal Cancer Patients Are Associated with Irinotecan-Induced Delayed Diarrhea. J. Pharmacol. Exp. Ther..

[6] Mathijssen R.H.J., Loos W.J., Verweij J., Sparreboom A. (2002). Pharmacology of Topoisomerase I Inhibitors Irinotecan (CPT-11) and Topotecan. Curr. Cancer Drug Targets.

[7] Liu L.F., Desai S.D., Li T.K., Mao Y., Sun M., Sim S.P. (2000). Mechanism of Action of Camptothecin. Ann. N. Y. Acad. Sci..

[8] Bao X., Wu J., Kim S., LoRusso P., Li J. (2019). Pharmacometabolomics Reveals Irinotecan Mechanism of Action in Cancer Patients. J. Clin. Pharmacol..

[9] Casadó A., Sagristá M.L., Mora Giménez M. (2018). A Novel Microfluidic Liposomal Formulation for the Delivery of the SN-38 Camptothecin: Characterization and in Vitro Assessment of Its Cytotoxic Effect on Two Tumor Cell Lines. Int. J. Nanomed..

[10] Yue B., Gao R., Wang Z., Dou W. (2021). Microbiota-Host-Irinotecan Axis: A New Insight Toward Irinotecan Chemotherapy. Front. Cell. Infect. Microbiol..

[11] Unrug-Bielawska K., Earnshaw D., Cybulska-Lubak M., Kaniuga E., Sandowska-Markiewicz Z., Statkiewicz M., Rumienczyk I., Dąbrowska M., Kocik-Krol J., Klimkiewicz K. (2024). Therapeutic Responses to Two New SN-38 Derivatives in Colorectal Cancer Patient-Derived Xenografts and Respective 3D In Vitro Cultures. Anticancer Res..

[12] Lensu S., Pekkala S. (2021). Gut Microbiota, Microbial Metabolites and Human Physical Performance. Metabolites.

[13] Perillo F., Amoroso C., Strati F., Giuffrè M.R., Díaz-Basabe A., Lattanzi G., Facciotti F. (2020). Gut Microbiota Manipulation as a Tool for Colorectal Cancer Management: Recent Advances in Its Use for Therapeutic Purposes. Int. J. Mol. Sci..

[14] Alexander J.L., Wilson I.D., Teare J., Marchesi J.R., Nicholson J.K., Kinross J.M. (2017). Gut Microbiota Modulation of Chemotherapy Efficacy and Toxicity. Nat. Rev. Gastroenterol. Hepatol..

[15] Wei L., Wen X.-S., Xian C.J. (2021). Chemotherapy-Induced Intestinal Microbiota Dysbiosis Impairs Mucosal Homeostasis by Modulating Toll-like Receptor Signaling Pathways. Int. J. Mol. Sci..

[16] Li S., Zhu S., Yu J. (2024). The Role of Gut Microbiota and Metabolites in Cancer Chemotherapy. J. Adv. Res..

[17] Cybulska M., Olesinski T., Goryca K., Paczkowska K., Statkiewicz M., Kopczynski M., Grochowska A., Unrug-Bielawska K., Tyl-Bielicka A., Gajewska M. (2018). Challenges in Stratifying the Molecular Variability of Patient-Derived Colon Tumor Xenografts. BioMed Res. Int..

[18] Zeber-Lubecka N., Kulecka M., Jagiełło-Gruszfeld A., Dąbrowska M., Kluska A., Piątkowska M., Bagińska K., Głowienka M., Surynt P., Tenderenda M. (2024). Breast Cancer but Not the Menopausal Status Is Associated with Small Changes of the Gut Microbiota. Front. Oncol..

[19] Kulecka M., Czarnowski P., Bałabas A., Turkot M., Kruczkowska-Tarantowicz K., Żeber-Lubecka N., Dąbrowska M., Paszkiewicz-Kozik E., Walewski J., Ługowska I. (2024). Microbial and Metabolic Gut Profiling across Seven Malignancies Identifies Fecal Faecalibacillus Intestinalis and Formic Acid as Commonly Altered in Cancer Patients. Int. J. Mol. Sci..

[20] Callahan B.J., McMurdie P.J., Rosen M.J., Han A.W., Johnson A.J.A., Holmes S.P. (2016). DADA2: High-Resolution Sample Inference from Illumina Amplicon Data. Nat. Methods.

[21] Schloss P.D., Westcott S.L., Ryabin T., Hall J.R., Hartmann M., Hollister E.B., Lesniewski R.A., Oakley B.B., Parks D.H., Robinson C.J. (2009). Introducing Mothur: Open-Source, Platform-Independent, Community-Supported Software for Describing and Comparing Microbial Communities. Appl. Environ. Microbiol..

[22] Quast C., Pruesse E., Yilmaz P., Gerken J., Schweer T., Yarza P., Peplies J., Glöckner F.O. (2013). The SILVA Ribosomal RNA Gene Database Project: Improved Data Processing and Web-Based Tools. Nucleic Acids Res..

[23] lmerTest lmerTest-Package: lmerTest: Tests in Linear Mixed Effects Models. https://rdrr.io/cran/lmerTest/man/lmerTest-package.html.

[24] Zhou H., He K., Chen J., Zhang X. (2022). LinDA: Linear Models for Differential Abundance Analysis of Microbiome Compositional Data. Genome Biol..

[25] Forsgård R.A., Marrachelli V.G., Korpela K., Frias R., Collado M.C., Korpela R., Monleon D., Spillmann T., Österlund P. (2017). Chemotherapy-Induced Gastrointestinal Toxicity Is Associated with Changes in Serum and Urine Metabolome and Fecal Microbiota in Male Sprague-Dawley Rats. Cancer Chemother. Pharmacol..

[26] Crossland N.A., Beck S., Tan W.Y., Lo M., Mason J.B., Zhang C., Guo W., Crott J.W. (2023). Fecal Microbiota Transplanted from Old Mice Promotes More Colonic Inflammation, Proliferation, and Tumor Formation in Azoxymethane-Treated A/J Mice than Microbiota Originating from Young Mice. Gut Microbes.

[27] André T., Boni C., Mounedji-Boudiaf L., Navarro M., Tabernero J., Hickish T., Topham C., Zaninelli M., Clingan P., Bridgewater J. (2004). Oxaliplatin, Fluorouracil, and Leucovorin as Adjuvant Treatment for Colon Cancer. N. Engl. J. Med..

[28] Song Y., Wang X., Lu X., Wang T. (2024). Exposure to Microcystin-LR Promotes Colorectal Cancer Progression by Altering Gut Microbiota and Associated Metabolites in APC^min/+^ Mice. Toxins.

[29] Newsome R.C., Gharaibeh R.Z., Pierce C.M., Da Silva W.V., Paul S., Hogue S.R., Yu Q., Antonia S., Conejo-Garcia J.R., Robinson L.A. (2022). Interaction of Bacterial Genera Associated with Therapeutic Response to Immune Checkpoint PD-1 Blockade in a United States Cohort. Genome Med..

[30] Chen K.J., Chen Y.L., Ueng S.H., Hwang T.L., Kuo L.M., Hsieh P.W. (2021). Neutrophil Elastase Inhibitor (MPH-966) Improves Intestinal Mucosal Damage and Gut Microbiota in a Mouse Model of 5-Fluorouracil–Induced Intestinal Mucositis. Biomed. Pharmacother..

[31] Wang X., Zhu B., Hua Y., Sun R., Tan X., Chang X., Tang D., Gu J. (2024). Astragalus Mongholicus Bunge and Curcuma Aromatica Salisb. Modulate Gut Microbiome and Bile Acid Metabolism to Inhibit Colon Cancer Progression. Front. Microbiol..

[32] Karami P., Goli H.R., Abediankenari S., Chandani S.R., Jafari N., Ghasemi M., Ahanjan M. (2023). Anti-Tumor Effects of *Bacteroides Fragilis* and *Bifidobacterium Bifidum* Culture Supernatants on Mouse Breast Cancer. Gene Rep..

[33] Ma W., Zhang L., Chen W., Chang Z., Tu J., Qin Y., Yao Y., Dong M., Ding J., Li S. (2024). Microbiota Enterotoxigenic *Bacteroides Fragilis* -Secreted BFT-1 Promotes Breast Cancer Cell Stemness and Chemoresistance through Its Functional Receptor NOD1. Protein Cell.

[34] Lee Y.K., Mehrabian P., Boyajian S., Wu W.-L., Selicha J., Vonderfecht S., Mazmanian S.K. (2018). The Protective Role of *Bacteroides Fragilis* in a Murine Model of Colitis-Associated Colorectal Cancer. mSphere.

[35] Hills R., Pontefract B., Mishcon H., Black C., Sutton S., Theberge C. (2019). Gut Microbiome: Profound Implications for Diet and Disease. Nutrients.

[36] Niu H., Zhou M., Zogona D., Xing Z., Wu T., Chen R., Cui D., Liang F., Xu X. (2024). Akkermansia Muciniphila: A Potential Candidate for Ameliorating Metabolic Diseases. Front. Immunol..

[37] Sheng Q.-S., He K.-X., Li J.-J., Zhong Z.-F., Wang F.-X., Pan L.-L., Lin J.-J. (2020). Comparison of Gut Microbiome in Human Colorectal Cancer in Paired Tumor and Adjacent Normal Tissues. OncoTargets Ther..

[38] Liu X., Cui S., Zhang L., Wu S., Feng C., Liu B., Yang H. (2024). Gut Microbiota Affects the Activation of STING Pathway and Thus Participates in the Progression of Colorectal Cancer. World J. Surg. Oncol..

[39] Lin X.B., Dieleman L.A., Ketabi A., Bibova I., Sawyer M.B., Xue H., Field C.J., Baracos V.E., Gänzle M.G. (2012). Irinotecan (CPT-11) Chemotherapy Alters Intestinal Microbiota in Tumour Bearing Rats. PLoS ONE.

[40] Wang Y., Sun L., Chen S., Guo S., Yue T., Hou Q., Feng M., Xu H., Liu Y., Wang P. (2019). The Administration of Escherichia Coli Nissle 1917 Ameliorates Irinotecan–Induced Intestinal Barrier Dysfunction and Gut Microbial Dysbiosis in Mice. Life Sci..

[41] Stringer A.M., Gibson R.J., Logan R.M., Bowen J.M., Yeoh A.S.J., Keefe D.M.K. (2008). Faecal Microflora and Beta-Glucuronidase Expression Are Altered in an Irinotecan-Induced Diarrhea Model in Rats. Cancer Biol. Ther..

[42] Stringer A.M., Gibson R.J., Bowen J.M., Logan R.M., Ashton K., Yeoh A.S.J., Al-Dasooqi N., Keefe D.M.K. (2009). Irinotecan-induced Mucositis Manifesting as Diarrhoea Corresponds with an Amended Intestinal Flora and Mucin Profile. Int. J. Exp. Pathol..

[43] Stringer A.M., Gibson R.J., Logan R.M., Bowen J.M., Yeoh A.S.-J., Burns J., Keefe D.M.K. (2007). Chemotherapy-Induced Diarrhea Is Associated with Changes in the Luminal Environment in the DA Rat. Exp. Biol. Med..

[44] Wang L., Wang R., Wei G., Wang S., Du G. (2020). Dihydrotanshinone Attenuates Chemotherapy-Induced Intestinal Mucositis and Alters Fecal Microbiota in Mice. Biomed. Pharmacother..

[45] Mekonnen S.A., Merenstein D., Fraser C.M., Marco M.L. (2020). Molecular Mechanisms of Probiotic Prevention of Antibiotic-Associated Diarrhea. Curr. Opin. Biotechnol..

[46] Fusco W., Lorenzo M.B., Cintoni M., Porcari S., Rinninella E., Kaitsas F., Lener E., Mele M.C., Gasbarrini A., Collado M.C. (2023). Short-Chain Fatty-Acid-Producing Bacteria: Key Components of the Human Gut Microbiota. Nutrients.

[47] Frampton J., Murphy K.G., Frost G., Chambers E.S. (2020). Short-Chain Fatty Acids as Potential Regulators of Skeletal Muscle Metabolism and Function. Nat. Metab..

[48] Oliphant K., Allen-Vercoe E. (2019). Macronutrient Metabolism by the Human Gut Microbiome: Major Fermentation by-Products and Their Impact on Host Health. Microbiome.

[49] Macfarlane S., Quigley M.E., Hopkins M.J., Newton D.F., Macfarlane G.T. (1998). Polysaccharide Degradation by Human Intestinal Bacteria during Growth under Multi-Substrate Limiting Conditions in a Three-Stage Continuous Culture System. FEMS Microbiol. Ecol..

[50] LeBlanc J.G., Chain F., Martín R., Bermúdez-Humarán L.G., Courau S., Langella P. (2017). Beneficial Effects on Host Energy Metabolism of Short-Chain Fatty Acids and Vitamins Produced by Commensal and Probiotic Bacteria. Microb. Cell Fact..

[51] Markowiak-Kopeć P., Śliżewska K. (2020). The Effect of Probiotics on the Production of Short-Chain Fatty Acids by Human Intestinal Microbiome. Nutrients.

[52] Lin X.B., Farhangfar A., Valcheva R., Sawyer M.B., Dieleman L., Schieber A., Gänzle M.G., Baracos V. (2014). The Role of Intestinal Microbiota in Development of Irinotecan Toxicity and in Toxicity Reduction through Dietary Fibres in Rats. PLoS ONE.

[53] Hueso T., Ekpe K., Mayeur C., Gatse A., Joncquel-Chevallier Curt M., Gricourt G., Rodriguez C., Burdet C., Ulmann G., Neut C. (2020). Impact and Consequences of Intensive Chemotherapy on Intestinal Barrier and Microbiota in Acute Myeloid Leukemia: The Role of Mucosal Strengthening. Gut Microbes.

[54] Ziemons J., Aarnoutse R., Heuft A., Hillege L., Waelen J., De Vos-Geelen J., Valkenburg-van Iersel L., Van Hellemond I.E.G., Creemers G.-J.M., Baars A. (2023). Fecal Levels of SCFA and BCFA during Capecitabine in Patients with Metastatic or Unresectable Colorectal Cancer. Clin. Exp. Med..

[55] Bai Z., Liu Y., Zhao Y., Yan R., Yang L., Ma H., Wang J., Wang T., Li Y., Zhang G. (2023). Aspirin Ameliorates Atherosclerotic Immuno-Inflammation through Regulating the Treg/Th17 Axis and CD39–CD73 Adenosine Signaling via Remodeling the Gut Microbiota in ApoE^−/−^ Mice. Int. Immunopharmacol..

[56] Encarnação J.C., Pires A.S., Amaral R.A., Gonçalves T.J., Laranjo M., Casalta-Lopes J.E., Gonçalves A.C., Sarmento-Ribeiro A.B., Abrantes A.M., Botelho M.F. (2018). Butyrate, a Dietary Fiber Derivative That Improves Irinotecan Effect in Colon Cancer Cells. J. Nutr. Biochem..

[57] Son M.-Y., Cho H.-S. (2023). Anticancer Effects of Gut Microbiota-Derived Short-Chain Fatty Acids in Cancers. J. Microbiol. Biotechnol..

[58] Maria R.M., Altei W.F., Selistre-de-Araujo H.S., Colnago L.A. (2017). Effects of Doxorubicin, Cisplatin, and Tamoxifen on the Metabolic Profile of Human Breast Cancer MCF-7 Cells As Determined by 1H High-Resolution Magic Angle Spinning Nuclear Magnetic Resonance. Biochemistry.

[59] Wang W., Zou W. (2020). Amino Acids and Their Transporters in T Cell Immunity and Cancer Therapy. Mol. Cell.

[60] Wang W., Kryczek I., Dostál L., Lin H., Tan L., Zhao L., Lu F., Wei S., Maj T., Peng D. (2016). Effector T Cells Abrogate Stroma-Mediated Chemoresistance in Ovarian Cancer. Cell.

[61] Tintelnot J., Xu Y., Lesker T.R., Schönlein M., Konczalla L., Giannou A.D., Pelczar P., Kylies D., Puelles V.G., Bielecka A.A. (2023). Microbiota-Derived 3-IAA Influences Chemotherapy Efficacy in Pancreatic Cancer. Nature.

[62] Zidi O., Souai N., Raies H., Ben Ayed F., Mezlini A., Mezrioui S., Tranchida F., Sabatier J.-M., Mosbah A., Cherif A. (2021). Fecal Metabolic Profiling of Breast Cancer Patients during Neoadjuvant Chemotherapy Reveals Potential Biomarkers. Molecules.

[63] Deng Y., Hou X., Fang Q., Wang H., Li X., Hu Z., Liu Z., Fan L., Liu Y., Fu Z. (2025). High-Salt Diet Decreases FOLFOX Efficacy via Gut Bacterial Tryptophan Metabolism in Colorectal Cancer. Mol. Med..

[64] Ribeiro R.A., Wanderley C.W.S., Wong D.V.T., Mota J.M.S.C., Leite C.A.V.G., Souza M.H.L.P., Cunha F.Q., Lima-Júnior R.C.P. (2016). Irinotecan- and 5-Fluorouracil-Induced Intestinal Mucositis: Insights into Pathogenesis and Therapeutic Perspectives. Cancer Chemother. Pharmacol..

[65] Chang C.-W., Lee H.-C., Li L.-H., Chiang Chiau J.-S., Wang T.-E., Chuang W.-H., Chen M.-J., Wang H.-Y., Shih S.-C., Liu C.-Y. (2020). Fecal Microbiota Transplantation Prevents Intestinal Injury, Upregulation of Toll-Like Receptors, and 5-Fluorouracil/Oxaliplatin-Induced Toxicity in Colorectal Cancer. Int. J. Mol. Sci..

[66] Soufizadeh P., Mansouri V., Ahmadbeigi N. (2024). A Review of Animal Models Utilized in Preclinical Studies of Approved Gene Therapy Products: Trends and Insights. Lab. Anim. Res..

[67] Anonymous (2024). What Differentiates Clinical Trial Statistics from Preclinical Methods and Why Robust Approaches Matter. Nat. Commun..

